# The effect of selenium on antioxidant system in aquaculture animals

**DOI:** 10.3389/fphys.2023.1153511

**Published:** 2023-04-26

**Authors:** Zi-Meng Li, Xiu-Li Wang, Xiao-Min Jin, Jia-Qiang Huang, Lian-Shun Wang

**Affiliations:** ^1^ The Key Laboratory of Pufferfish Breeding and Culture in Liaoning Province, Dalian Ocean University, Dalian, China; ^2^ College of Fisheries an Life, Dalian Ocean University, Dalian, Liaoning, China; ^3^ Hebei Key Laboratory of Ocean Dynamics Resources and Environments, Hebei Normal University of Science and Technology, Qinhuangdao, China; ^4^ Department of Nutrition and Health, China Agricultural University, Beijing, China

**Keywords:** selenium, aquatic animals, antioxidant, oxidative stress, selenoprotein

## Abstract

There will be generated some adverse conditions in the process of acquculture farming with the continuous improvement of the intensive degree of modern aquaculture, such as crowding stress, hypoxia, and malnutrition, which will easily lead to oxidative stress. Se is an effective antioxidant, participating and playing an important role in the antioxidant defense system of fish. This paper reviews the physiological functions of selenoproteins in resisting oxidative stress in aquatic animals, the mechanisms of different forms of Se in anti-oxidative stress in aquatic animals and the harmful effects of lower and higher levels of Se in aquaculture. To summarize the application and research progress of Se in oxidative stress in aquatic animals and provide scientific references for its application in anti-oxidative stress in aquaculture.

## 1 Introduction

Selenium (Se) is an essential micronutrient that maintains growth, health and development in human and animals (Hatfield et al., 2012). Se exists in nature is available as inorganic form and organic form. In addition, there is also a new type of synthetic nano form. The biological functions of Se include promoting the growth and development of animals, improving the antioxidant capacity of the body, avoiding oxidative stress, enhancing immunity, improving the health of the body, and are widely used in the growth and development of aquaculture animals (Wischhusen et al., 2020; Khan et al., 2017; Jingyuan et al., 2020). However, Se cannot be directly digested and absorbed by organisms. Se appears functions in the form of selenoprotein and exerts multiple and complex effects (Wrobel et al., 2016).

There will be generated some adverse conditions in the process of aquaculture farming with the continuous improvement of the intensive degree of modern aquaculture, such as crowding stress, hypoxia, and malnutrition. Meanwhile, that will promote the production of excessive reactive oxygen species (ROS), change the balance of oxidation and reduction in cells and will lead to oxidative stress (Rider et al., 2009; Abdel Tawwab et al., 2019). Once oxidative stress occurs, it may lead to the dynamic imbalance of free radical stability, which ultimately the fish poor growth and decline the meat quality (Chadio et al., 2015). The damage of oxidative stress in production is increasingly prominent, so it is urgent to solve the problem of oxidative stress in aquaculture animals.

In order to prevent oxidative damage caused by adverse conditions, aquaculture animals must have an effective antioxidant system, such as endogenous free radical scavenging enzymes and exogenous antioxidants (Ighodaro and Akinloye, 2018). Se is just a extra-effective antioxidant and plays an important role in the antioxidant defense mechanism of fish. It protects the cell membrane from oxidative damage through regulate glutathione peroxidase (GPX) activity (Hamilton, 2004). Se can decompose and clear peroxides, thus preventing the accumulation of reactive oxygen species and free radicals such as hydrogen peroxide (H_2_O_2_) in the body tissues (Zuo et al., 2019), enhancing resistance to inflammation, maintaining immune function and stabilizing the internal environment (Wande et al., 2020). However, dietary Se deficiency will reduce the expression of selenoproteins, increase the peroxidation reaction of lipids, proteins, DNA and then pose a threat to the health of the body (Rao et al., 2020). Excessive Se will also produce toxic side effects on the body, leading to liver damage, physiological function decline, immune capacity reduction, and even death (Chadio et al., 2015).

At present, the research on Se antioxidant stress has involved inorganic Se (such as sodium selenite), organic Se (such as yeast Se, selenomethionines) and nano Se. In the study of the effects of sodium selenite and *lactobacillus* on the growth and immune response of rainbow trout, the results showed that the use of sodium selenite and *lactobacillus* as feed additives could improve the growth and metabolism. Also, the effect of *lactobacillus* on the natural immune response of rainbow trout was also enhanced under the effect of selenite (Kousha et al., 2020). Another study found that, the mortality rate and malondialdehyde (MDA) level of grass carp fed a high-fat diet supplemented with 0.3 and 0.6 mg/kg nano Se was significantly reduced after hypoxia stress, indicating that nano Se can alleviate lipid peroxidation, thus alleviating the oxidative damage of grass carp under hypoxia stress (Yu et al., 2020). Liu et al. (2017) showed that 0.4 mg/kg yeast Se significantly increased the activity of catalase (CAT) and GPX in the liver of blunt snout bream and significantly decreased the activity of glutathione reductase (GR) and the content of MDA, showing strong antioxidant capacity.

## 2 The role of selenoproteins in the antioxidant system of aquatic animals

In general, Se sources in animals will first be converted into selenides for play on organisms and then will be converted into proteins containing selenocysteine (Sec), that is, selenoprotein which then will be absorbed and used by organisms (Yoboue et al., 2018). During the process of fish growth, any pressure condition will directly or indirectly cause oxidative stress, leading to the level of intracellular ROS related to oxidative stress increasing, causing destructive effects on lipids, proteins, and DNA (Martínez Álvarez et al., 2005; Zenteno Savín et al., 2006). The lack of balance between the production of ROS and the antioxidant defense system of animals will lead to DNA hydroxylation, protein denaturation, lipid peroxidation, cell apoptosis, ultimately lead to cell damage (Yang et al., 2016). As an effective antioxidant, Se plays an important role in the antioxidant defense system of aquaculture animals. It can maintain balance in the body and antioxidant system by generating and removing ROS (Steinbrenner and Sies, 2009).

It has been found that the thioredoxin reductase system and glutathione peroxidase system play an important role in cellular oxidative stress defense (Lee et al., 2012; Benfeitas et al., 2014). The main function of GPX in mammals is to eliminate excessive free radicals in cells, including superoxide anions (O^2-^), H_2_O_2_, and hydroxyl radicals (OH^−^). Then through reduce peroxides to corresponding alcohols, prevent lipid peroxidation, and regulate the balance of redox in cells (Yoboue et al., 2018; Rocca et al., 2018). The Thioredoxin system is one of the main redox buffer systems in the body, which plays a key role in the transduction of redox signals (Canter et al., 2021; Shchedrina et al., 2010). In addition, the endoplasmic reticulum (ER) is one of the important places where redox reactions occur in cells, which can produce H_2_O_2_ and ROS (Varlamova, 2018). SELENOF, Recombinant Deiodinase, Iodothyronine, Type II (DIO2), SELENOS, SELENON, SELENOK, SELENOM, and SELENOT are considered to be seven endoplasmic reticulum resident proteins with oxidoreductase activity and can participate in a series of processes, such as the regulation of endoplasmic reticulum redox state, endoplasmic reticulum stress and intracellular Ca^2+^ homeostasis (Malandrakis et al., 2014; Pitts and Hoffmann, 2017; Drew et al., 2008; Addinsall et al., 2018; Rathore et al., 2021; Xu et al., 2022) Specific selenoprotein involved in redox reaction are shown in Table 1.

**TABLE 1:** Antioxidant functions of releated selenoproteins.

Type	Aquatic animal species	Antioxidant function	References
*GPX1*	*Danio rerio, Sparus aurata*	Neutralize reactive oxygen species and improve oxidative stress	Malandrakis et al. (2014), Rathore et al. (2021)
*GPX2*	*Oreochromis niloticus*	Reduction of H_2_O_2_	Xu et al. (2022)
*GPX3*	*Pelteobagrus fulvidraco*	Affects oxidative signaling	Guderley et al. (2003)
*GPX4*	*Gadus morhua, Thunnus maccoyi, Coho salmon*	Protection of vertebrate olfactory system from oxidative cell damage by lipid peroxidation	Solovyev (2015), Thompson et al. (2010), Wang et al. (2012)
*TXNRD1*	*Pelteobagrus fulvidraco*	Reduction of H_2_O_2_ and oxidative stress and regulation of redox-sensitive transcription factors that control cellular transcriptional machinery	Chernorudskiy et al. (2020)
*TXNRD2*	*Pelteobagrus fulvidraco*	Reduction of thioredoxin to protect cells from oxidation	Guderley et al. (2003)
*SELENON*	*Pelteobagrus fulvidraco*	Regulates calcium homeostasis and maintains redox activity	Saito (2021)
*SELENOT*	*Pelteobagrus fulvidraco*	Control protein processing in the ER and maintain ER homeostasis	Guderley et al. (2003)
*SELENOP*	*Zerbfish*	Reduction of phospholipid hydroperoxides	Zhang et al. (2022)
*SELENOF*	*Pelteobagrus fulvidraco*	Regulation of endoplasmic reticulum stress, regulation of lipid and glucose metabolism	Zhang et al. (2021)
*SELENOM*	*Pelteobagrus fulvidraco*	Regulation of ER stress and calcium homeostasis	Wang et al. (2018a)
*SELENOK*	*Oreochromis niloticus*	Regulation of Ca^2+^ metabolism in gills and mitigation of ER stress	Zhang et al. (2021)
*SELENOL*	*Oreochromis niloticus*	With thioredoxin structure, with redox function	Zhang et al. (2021)
*SELENOS*	*Oreochromis, niloticus, Pelteobagrus fulvidraco*	Regulation of lipid metabolism and endoplasmic reticulum stress	Zhang et al. (2021); Wang et al. (2018a)
*SELENOW*	*Oncorhynchus mykiss*	Protects skeletal muscle from inflammatory damage	Castellano et al. (2005)
*SELENOJ*	*Oreochromis niloticus*	Affects cellular resistance to stress	He et al. (2022)
*SELENOU*	*Ctenopharyngodon idella*	Regulation of lipid metabolism	Bityutskyy et al. (2020)
*MSRB1*	*Salmon fish*	Regulates the inflammatory response	Penglase et al. (2014)
*SPS1*	*Oreochromis niloticus*	Affects gill ion exchange and has oxidative stress defense	Rathore et al. (2021)

ER, endoplasmic reticulum; GPX1, glutathione peroxidase 1; GPX2, glutathione peroxidase 2; GPX3, glutathione peroxidase 3; GPX4, glutathione peroxidase 4; TXNRD1, thioredoxin reductase 1; TXNRD2, thioredoxin reductase 2; SELENON, selenoprotein N; SELENOT, selenoprotein T; SELENOP: selenoprotein P; SELENOF: selenoprotein F; SELENOM: selenoprotein M; SELENOK: selenoprotein K; SELENOL, selenoprotein L; SELENOS, selenoprotein S; SELENOW, selenoprotein W; SELENOJ, selenoprotein J; SELENOU, selenoprotein U; MSRB1, methionine sulfoxide reductase B1; SPS1, selenophosphate synthetase 1.

## 3 Application of different forms of Se on antioxidant system of aquatic animals

In many studies, Se has been found that supplement oxidative stress caused by hypoxia, crowding, heavy metal pollution, intake oxidized oil and other pressure conditions (Schieber and Chandel, 2014; Chen et al., 2013). Oxidative stress is considered to be a major problem in aquaculture because it is reported that this phenomenon will occur naturally in most aquatic environments, especially in high-density intensive fish farming which will induce metabolic changes and produce a large number of reactive oxygen species (Zenteno Savín et al., 2006). In order to avoid the imbalance of ROS production, aquatic animals can reduce the formation of ROS or reduce the high reactivity of such compounds through antioxidant components which are produced by cells (endogenous sources) or obtained through diet (exogenous sources). A Lack of balance between ROS formation and the ability of organisms to process reactive oxygen will lead to oxidative stress (Wang et al., 2013; Ji et al., 2019). At present, many aquatic animals have been studied on the antioxidant system, and there is evidence that they can prevent or repair oxidative damage (Biller and Takahashi, 2018) (Table 2).

**TABLE 2:** Effects of different forms of Se on the antioxidant system of aquatic animals.

Type	Aquatic animal species	Antioxidant mechanism	References
Inorganic Se	*Rainbow Trout*	Inhibition of increased lipid peroxidation, inhibition of antioxidant transcript levels, inhibition of glutathione levels in the blood	Fontagné Dicharry et al. (2020)
	*Nile tilapia* (*Oreochromis niloticus L.*)	Inhibits lipid peroxidation in gills, increases non-protein thiol levels, participates in fish excretion	Giuliani Durigon et al. (2018)
	*Eriocheir sinensis*	Inhibits hepatopancreas lipid peroxide production and enhances GPX activity	Qiang et al. (2020)
	*Micropterus salmoide*	Enhancement of hepatic GPX and CAT activity	Chen et al. (2013)
Organic Se	*Litopenaeus vannamei*	Catalyzing the dismutation of superoxide radicals into H_2_O_2_ and oxygen, and the conversion of H_2_O_2_ into water, protecting cell membranes from oxidative damage, participating in polyunsaturated fatty acid metabolism	Yu et al. (2022)
	*Acanthopagrus schlegelii*	Regulation of hepatic lipid metabolism, enhancement of hepatic GPX activity	Wang et al. (2019)
	Gilthead seabream (*Sparus aurata*)	Inhibition of hepatic lipid peroxide production	Mechlaoui et al. (2019)
	*Rainbow Trout* (*Oncorhynchus mykiss*)	Inhibits muscle lipid peroxide production, enhances muscle GPX activity, inhibits SOD and CAT activity	Wang et al. (2018a)
Nano-Se	*Nile tilapia* (*Oreochromis niloticus*)	Affects fish excretion and gill ion exchange	Rathore et al. (2021)
	*Grass carp* (*Ctenopharyngodon Idella*)	Regulation of Nrf_2_ signaling and selective expression of antioxidant enzyme genes	Yu et al. (2020)
	*Cyprinus carpio*	Enhance hepatic GPX, SOD and CAT activities and inhibit hepatic peroxide production	Saffari et al. (2017)

SOD, superoxide dismutase.

## 4 Harmful effects of Se deficiency and excess on antioxidant function in aquatic animals

After these environmental pressures, Se may have protective effects on the pathology related to oxidative stress of aquatic animals. It is particularly important for intensive aquaculture animals, because environmental pressures may cause significant losses to fish growers. The demand for Se will increase after suffering environmental pressures, and supplementation is a necessary condition to meet the needs of fish growth (Ashouri et al., 2015). Se can act as a dietary supplement to effectively improve the oxidation status of aquaculture animals. Although, dietary Se application in aquatic animal feed is considered to have a narrow intake range for high concentration may be toxic (Nasir et al., 2015) and lack may have adverse effects on fish health, leading to tissue damage and physiological function weakening (Khan and Bano, 2016). Therefore, their safety in aquaculture animals is controversial.

### 4.1 Harmful effects of selenium excess

Se is an essential micronutrient required for fish, which plays an important role in the antioxidant system and also affects the lipid metabolism, sugar metabolism and amino acid metabolism of aquatic animals (Yoboue et al., 2018; Fontagné Dicharry et al., 2015; Abdel Tawwab et al., 2007). Although Se supplementation in aquatic feed can alleviate the oxidation state of cultured animals, but excessive Se content will also have some toxic effects (Jobling, 2012). When the Se content in the aquatic feed is slightly higher than the demand, it will have other adverse effects including oxidative stress, cytotoxicity and genotoxicity (Gobi et al., 2018). If the fish is exposed to excessive Se, the degree of oxidative stress in the body can be significantly increased, then cause oxidative stress. Glutathione (GSH) is largely used to maintain the homeostasis of ROS in the body, can cause an imbalance in the antioxidant system (Atencio et al., 2009). In Atlantic salmon, higher Se levels (at least 15 mg/kg in diet) can lead to oxidative stress and change the lipid metabolism of organic and inorganic Se (Berntssen et al., 2017). In addition, in Acipenser sturgeon (*Acipenser transmontanus*), excessive Se in the diet leads to vacuolar degeneration of cells and necrosis of the liver (Tashjian et al., 2006). In a word, the mechanism of the negative effect of high Se intake on the antioxidant system of aquatic animals is still unclear.

### 4.2 Harmful effects of Se deficiency

The necessity and demand for dietary Se have been estimated in various fish (Khan et al., 2017). Although Se poisoning may cause multiple damages to fish, the effect of dietary Se deficiency in fish cannot be ignored. It has been reported that Se deficiency is closely related to the occurrence of many diseases in aquatic animals and the impairment of physiological functions under various conditions (Zheng et al., 2018; Wang et al., 2018). Se deficiency can inhibit organs growth and reduce immune functions, thus leading to many inflammatory diseases (Zheng et al., 2018; Hofstee et al., 2017). Se has been proved to be a protective trace element in the liver of many animals. Se deficiency can lead to immune deficiency, inhibit fish growth and induce oxidative stress in the liver (Zheng et al., 2018; Byron and Santolo, 2018). Oxidative stress can stimulate liver inflammation, thereby aggravating liver injury (Mukhopadhyay et al., 2018). In a research on the mechanism of carp liver inflammation, dietary Se deficiency can lead to Se deficiency in blood and liver, leading to overexpression of heat shock protein HSP60 in the liver, aggravating and forming inflammatory damage to the liver (Gao et al., 2019). Se deficiency also impairs the structural integrity of the head kidney, spleen and skin of juvenile grass carp, leads to oxidative damage and aggravating cell apoptosis due to downregulate the activities of antioxidant enzymes (SOD, CAT, GPX, GSTR, and GR) and the mRNA levels related to the signal transduction part (Zheng et al., 2018).

## 5 Effects of Se-rich fish on humans

Food is the main source of Se intake by the human. As like fish, Se is mainly involved in regulating human various physiological activities in the form of selenoproteins. Se deficiency or Se excess can have adverse effects on the human (Rayman, 2008). Fish is a kind of food with the highest natural Se content and tender flesh, rich in mineral elements, proteins and vitamins, which is the preferred ingredient for people to supplement trace elements through diet. Fish reared in a Se-rich water environment and fed with Se-rich feed can be processed and prepared into bioactive peptide foods such as fish collagen peptides. Eating Se-rich bioactive peptide foods can improve the absorption rate of Se in the body and Se supplement is more efficient (Xia et al., 2022).

Se can reduce the incidence rate of heart disease in humans, but ROS formed during oxidative stress can initiate lipid peroxidation, damage normal cell function, and lead to myocardial ischemia reperfusion (I/R) injury. Se enzyme and the whole thioredoxin system are directly reduce the content of lipid peroxide, protect vascular endothelial cells from oxidative damage, and inhibit Low Density Lipoprotein (LDL) oxidation (Furman et al., 2004; Venardos and Kaye, 2007). SELENOM may be able to alleviate disorders of lipid metabolism and high-fat diet (HFD)-mediated mitochondrial damage in NAFLD. SELENOM regulates Parkin-mediated mitophagy *via* the *AMPKα*_*1*_ *–MFN*_*2*_ signaling pathway, blockade of the *AMPKα*_*1*_ *–MFN*_*2*_ pathway and inhibit mitophagy. Moreover, SELENOM deletion inhibits the expression of FAO-related genes (*Ppara*, *Cpt1α*, *Cdh15*, *Acox1* and *Acadm*), and increases the expression of lipogenic genes (*Gpam*, *Plin1*, *Scd1*, *Lipe*, *Fasn*, *Acly,* and *Pparg*) (Cai et al., 2022). It has been shown that during wound healing some selenoproteins (GPX, SELENOS and SELENOP) bind together with produce effects in the inflammatory phase, such as antioxidant, inhibition of inflammatory cytokines, scavenging of peroxynitrite and enhancing immune function by increasing the survival of phagocytes during phagocytosis (Lei et al., 2009). Se is play a key role in reducing and preventing cancer caused by several carcinogens and inducing apoptosis of cancer cells (Tinggi, 2008). Se level is negatively correlated with human cancer risk. Genomic studies and animal models have indicated that Se intake influences the expression of selenoprotein genes and pathways key to colorectal carcinogenesis such as the antioxidant response, immune and inflammatory pathways (including *NF-kB* and *Nrf*_*2*_ signaling), the Wnt signaling pathway, protein synthesis pathway (Ye et al., 2021). Se also reduces the incidence of autoimmune thyroiditis and other related conditions, and the reduction of thyroid autoimmune effects is related to the role of GPX and thioredoxin reductase (TrxR) as antioxidant defense systems that scavenge ROS and excess H_2_O_2_ produced by thyroid cells during thyroid hormone synthesis, which can lead to thyroid cell necrosis and increased macrophage invasion in severe nutritional Se deficiency (Duntas, 2006).

## 6 Antioxidant mechanism of different Se forms in fish

**FIGURE 1:**
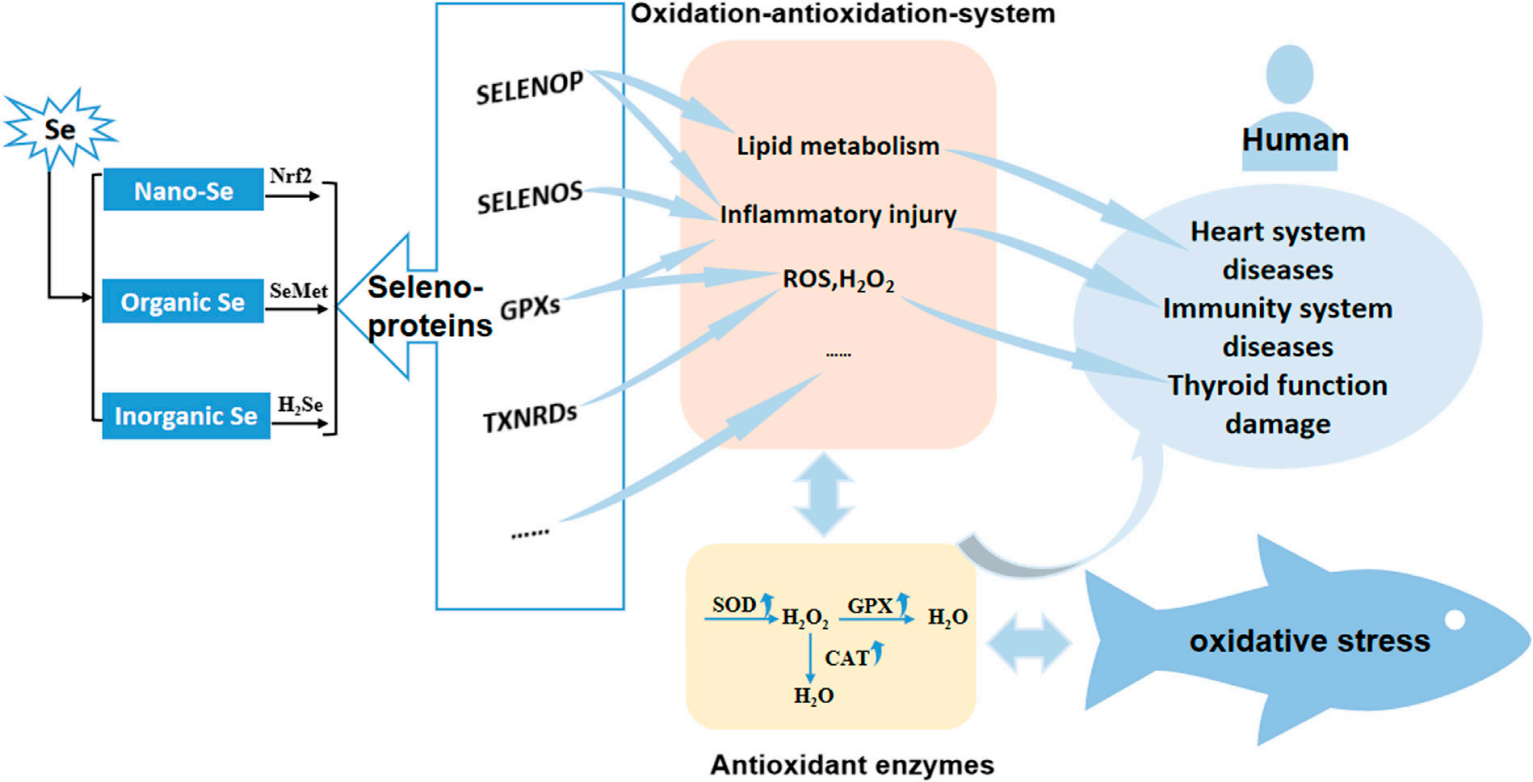
Antioxidant mechanism of different Se forms in fish. Nano-Se, Nano-Selenium; Organic Se, Organic Selenium; Inorganic Se, Inorganic Selenium; GPXs, glutathione peroxidase family; TXNRDs, thioredoxin reductase family.

In the intensive aquaculture system, cultured fish mainly obtain the selenium from the feed. There are three main forms of selenium additives: one is inorganic Se, which enters animals through passive diffusion in the form of Se^4+^, and is quickly reduced to selenides (H_2_Se) in the intestine; the second is organic Se, which is actively absorbed by fish in the form of amino acids, combines with plasma protein to form SeMet; the third is Nano-Se can activate Nrf_2_ signal transduction pathway to express genes related to antioxidant enzymes. No matter inorganic Se, organic Se or Nano-Se, it will be converted into selenoproteins to play a role after being absorbed by fish. Selenoproteins related to fish antioxidant mechanism include GPXs, TXNRDs, SELENOP, SELENOS, and other selenoproteins, which regulate lipid metabolism, inflammatory damage, ROS and H_2_O_2_ formation oxidative stress through antioxidant enzymes (SOD, GPX, and CAT) in fish antioxidant system. In addition, the regulation of selenium deficiency in the human antioxidant system will cause diseases of the heart system, immune system and thyroid function damage (Yu et al., 2020; Wischhusen et al., 2019) **(** Figure 1 **)**.

## 7 Conclusion

In summary, Se is an essential mineral element for antioxidant stress of aquatic animals and plays an important role in the antioxidant system of aquaculture. We can further study the most effective form of Se in the antioxidant system of aquatic animals, selectively prevent the oxidative stress of aquaculture animals, clarify the mechanism of selenoproteins in the antioxidant system of aquatic animals, and provide direction for the study of Se toxicity. Due to the various functions of selenoproteins, the mechanism of specific selenoprotein function and expression can be considered to prevent the oxidation state of aquatic animals. In the future, we can also compare the effects of Se on other organisms and study the mechanism of Se on the antioxidant system of aquatic animals. This will provide important information for the extensive application of antioxidant systems in aquaculture. This will provide important information for the extensive application of antioxidant systems in aquaculture.
